# Exposure to PCBs and *p,p*′-DDE and Human Sperm Chromatin Integrity

**DOI:** 10.1289/ehp.7252

**Published:** 2004-11-22

**Authors:** Anna Rignell-Hydbom, Lars Rylander, Aleksander Giwercman, B.A.G. Jönsson, Christian Lindh, Patrizia Eleuteri, Michele Rescia, Giorgio Leter, Eugenia Cordelli, Marcello Spano, Lars Hagmar

**Affiliations:** ^1^Department of Occupational and Environmental Medicine, Lund University Hospital, Lund, Sweden; ^2^Fertility Centre, Malmö University Hospital, Malmö, Sweden; ^3^Section of Toxicology and Biomedical Sciences, ENEA Casaccia Research Centre, Rome, Italy

**Keywords:** DDE, polychlorinated biphenyls, sperm chromatin integrity, sperm chromatin structure assay (SCSA)

## Abstract

Persistent organochlorine pollutants (POPs) such as polychlorinated biphenyls (PCBs) and dichlorodiphenyldichloroethylene (*p*,*p*′-DDE), the major metabolite of dichlorodiphenyltrichloroethane (DDT), are stable lipophilic compounds widely found in the environment and in the general population. They can enter the food chain, and their negative impact on male reproduction is currently under active scrutiny. To explore the hypothesis that environmental exposure to these compounds is associated with altered sperm chromatin structure integrity in human sperm, we conducted a study of 176 Swedish fishermen (with low and high consumption of fatty fish, a very important exposure source of POPs). We determined serum levels of 2,2′,4,4′,5,5′-hexachlorobiphenyl (CB-153) and *p*,*p*′-DDE, and we used the sperm chromatin structure assay (SCSA) to assess sperm DNA/chromatin integrity. When CB-153 serum levels (individual dose range, 39–1,460 ng/g lipid) were categorized into equally sized quintiles, we found an association with the DNA fragmentation index (%DFI). A significantly lower %DFI was found in the lowest CB-153 quintile (< 113 ng/g lipid) compared with the other quintiles; there was a similar tendency, although not statistically significant, between %DFI and *p*,*p*′-DDE. These results suggest that POP exposure may have a slight negative impact on human sperm chromatin integrity.

Polychlorinated biphenyls (PCBs), widely used in the past in cutting oils and lubricants and as an electric insulator, were restricted or totally banned in the 1970s in most developed countries, together with the insecticide dichlorodiphenyltrichloroethane (DDT). However, because of their high persistence to both biotic and abiotic degradation and their ability to bioaccumulate, these persistent organochlorine pollutants (POPs) continue to be a potential health hazard for the general population as they enter the food chain.

In Sweden, the consumption of fatty fish, such as salmon and herring, from the Baltic Sea off the Swedish east coast represents a major exposure source of PCBs, DDT, and its major metabolite, dichlorodiphenyldichloroethylene (*p*,*p*′-DDE). Fatty fish species from the Baltic Sea are much more contaminated with PCBs and *p*,*p*′-DDE than are corresponding fish from the Swedish west coast (Bergqvist et al. 1989). This is also the case with other POPs such as polychlorinated dibenzo-*p*-dioxins (PCDDs) and polychlorinated dibenzofurans (PCDFs). This was reflected in higher average plasma levels of dioxin-like POPs among east coast fishermen (290 pg/g lipid) than among west coast fishermen (139 pg/g lipid) and men from the general Swedish population (123 pg/g lipid) (Svensson et al. 1995).

PCBs are not a uniform group of compounds with similar biologic effects. Theoretically there are 209 PCB congeners, varying in the degree of chlorination and the position of chlorine atoms, which affect their stability and toxicity. In reality, fewer can be detected in the environment. The PCB congener 2,2′,4,4′,5,5′-hexachlorobiphenyl (CB-153) is a useful biomarker of dietary exposure to POPs because it correlates very well with both total PCB concentration (Gladen et al. 1999; Glynn et al. 2000; Grimvall et al. 1997), the 2,3,7,8-tetrachlorodibenzo-*p*-dioxin (TCDD) equivalent (TEQ), and the total POP derived TEQ (Brouwer et al. 1995; Gladen et al. 1999). Another relevant biomarker is the antiandrogenic compound *p*,*p*′-DDE, which is present in relatively high serum concentrations in men consuming fatty fish from the Baltic Sea (Sjodin et al. 2000).

Several studies on wildlife and laboratory animals have shown that exposure to PCBs and *p*,*p*′-DDE is capable of interfering with reproductive and endocrine functions (Cooke et al. 1996; Faqi et al. 1998; Fry 1995; Guillette et al. 1994, 1996; Hsu et al. 2003b; Kim 2001).

Human studies have shown that accidentally high exposures to PCBs and PCDFs have a negative effect on male reproductive function (Guo et al. 2000; Hsu et al. 2003a). Even more interesting is that lower exposure levels to PCBs, relevant for the general population in Western countries, have been associated with effects on sperm motility, sperm concentration, and total sperm count (Bush et al. 1986; Dallinga et al. 2002; Hauser et al. 2003a; Richthoff et al. 2003). Sperm motility seems to be especially vulnerable.

Sperm DNA integrity is essential for the accurate transmission of genetic information, and sperm chromatin abnormalities or DNA damage may result in male infertility (Agarwal and Said 2003). There are only a few human studies relating PCBs and *p*,*p*′-DDE levels in biologic fluids to sperm genetic integrity, which seems pivotal for the full expression of individual fertility potential (Hauser et al. 2003b; Rozati et al. 2002).

Among the variety of new methods to study sperm genetic integrity, sperm chromatin structure assay (SCSA) is considered as one of the most stable, robust, and objective (Evenson et al. 2002; Perreault et al. 2003). SCSA seems particularly fit for epidemiologic surveys because only a small (0.1 mL) amount of semen is needed for the analysis, and it can be frozen, stored, and assayed at the end of the study, minimizing interassay variation (Perreault et al. 2000). SCSA has been used in a number of epidemiologic studies among men exposed to pesticides, lead, styrene, solvents, and air pollution (Bonde et al. 2002; Kolstad et al. 1999; Larsen et al. 1998b; Perreault et al. 2000; Sanchez-Pena et al. 2004; Selevan et al. 2000) but not previously among POP-exposed subjects.

The aim of this study was to investigate whether serum levels of CB-153 and *p*,*p*′-DDE were associated with sperm chromatin damage assessed by SCSA.

## Materials and Methods

### Study population.

**Figure 1:**
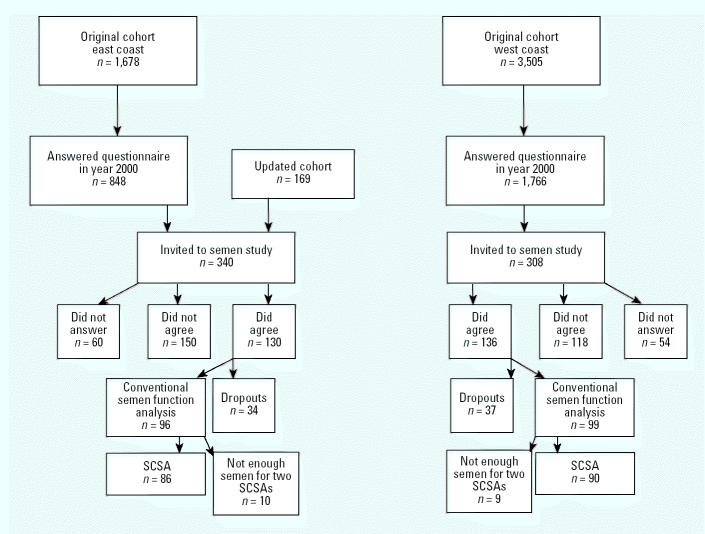
Flow chart for recruitment process of participants in the study.

Cohorts of fishermen from the Swedish east and west coasts were established in 1988 (Svensson et al. 1995). In 2000, a postal questionnaire, focused mainly on fracture incidence, was sent to 3,505 west coast fishermen and 1,678 east coast fishermen, born 1935 or later (Figure 1). The questionnaire included a question about whether the subjects were interested in more information on a study of semen function. Among the 2,614 subjects (east, *n* = 848; west, *n* = 1,766) who responded to this specific question, 479 (east, *n* = 171; west, *n* = 308) wanted more information about the semen study. We contacted these subjects and another 169 east coast fishermen who had become members of the east coast fishermen’s union after the closure of the cohorts. From the east coast, 130 of 340 men wanted to participate and gave their written informed consent. The corresponding figures from the west coast were 136 of 308. Thirty-four subjects from the east coast and 37 from the west coast were excluded for logistical reasons, changes of mind, sickness, or recent vasectomy during the field study period. In the end, 195 men participated in the semen study, and the results of standard semen analyses have been published previously (Rignell-Hydbom et al. 2004). Because of limited amounts of semen, samples from only 176 men could be used for SCSA.

### Nonparticipants.

The nonparticipants from the fishermen’s cohort had similar age distribution (median, 52 years; range, 29–67 years) as the participants in the present study [median, 48 years; range, 29–67 years]. The participants had on average 2.0 children. We do not have any directly comparable data for the nonparticipants, but a previous study showed that during 1973–1991 fishermen’s wives on average gave birth to 2.0 infants (Rylander et al. 1995). In addition, the body mass index (BMI) distributions and fraction of smokers were very similar among the participants and the nonparticipants.

### Questionnaire.

Approximately 2 weeks before telephone contact, a questionnaire regarding lifestyle and medical and reproductive history was sent out to the fishermen. In this way, the participants had time to get acquainted with the questions that they were interviewed on later. During the telephone contact, an agreement was reached on time and date for collection of semen and blood samples at the subject’s home. The participants received information on the procedures for collecting the semen samples both in verbal and written form. The study was approved by the Ethical Committee at Lund University.

### Mobile laboratory unit and semen and blood sampling.

A mobile laboratory unit was established for this study. The subjects were asked to keep 3 days’ abstinence time before sample collection (median, 3 days; range, 1–21 days), which took place in the participant’s homes. Immediate semen analyses were performed within 1 hr after ejaculation (Rignell-Hydbom et al. 2004). Two tubes with 200-μL aliquots of undiluted raw semen, collected 30 min after liquefaction, were directly put into a box with dry ice and shortly thereafter transferred into a freezer at −80°C. Venous blood samples were collected and centrifuged in the mobile laboratory, and sera were frozen at −80°C for later analysis.

### Sperm chromatin structure assay.

**Figure 2:**
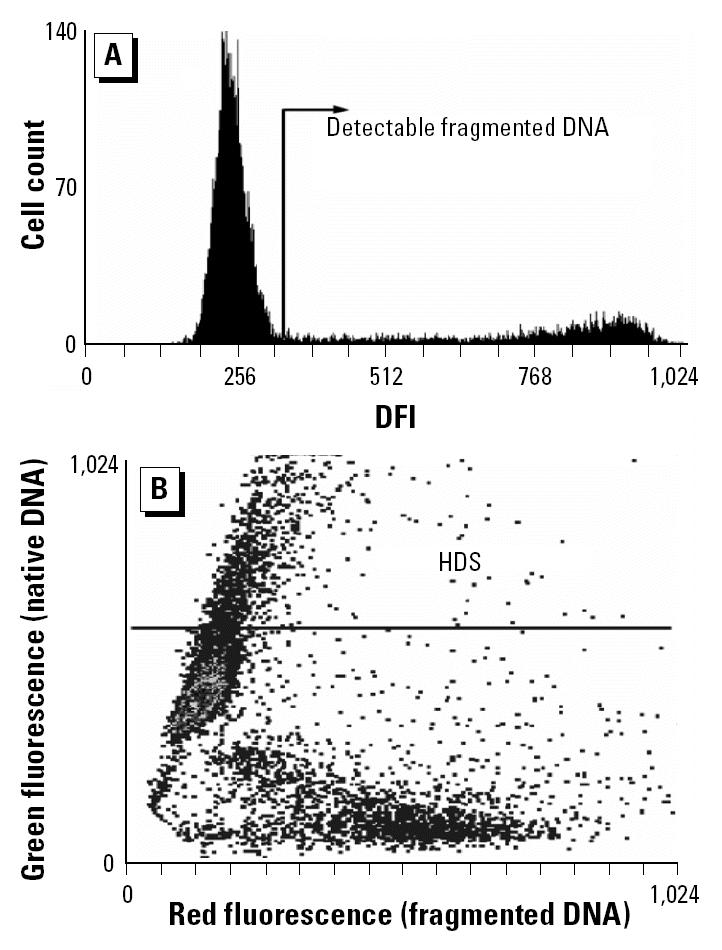
(*A*) Frequency distribution histogram of the DFI. The area located to the right of the main peak (which includes normal sperm with non-detectable DFI) represents the region where the sperm with detectable levels of fragmented DNA accumulate (%DFI). (*B*) SCSA scattergram of red (fragmented DNA, *x*-axis) versus green (double-stranded DNA, *y*-axis) fluorescence intensity of the same semen sample. Cytogram dots represent single spermatozoa with dual-parameter green and red fluorescence values acquired at 10-bit resolution (1,024 channels) on the flow cytometer. Debris (*B*, left) was excluded from the analysis. The region for calculating the fraction of immature sperm with HDS is indicated by the line. The line indicates the threshold for HDS (channel 550 on the *y*-axis).

The frozen samples were transported for flow cytometry (FCM) SCSA analysis to the Section of Toxicology and Biomedical Sciences, ENEA Casaccia, Rome, Italy. The samples were quickly thawed in a 37°C water bath and analyzed immediately. The SCSA was applied following the procedure described elsewhere (Evenson et al. 2002; Spano et al. 2000). A total of 1–2 × 10^6^ cells were treated with a detergent solution (pH 1.2) containing 0.1% Triton X-100, 0.15 M NaCl, and 0.08 N HCl for 30 sec and then stained with 6 mg/L of purified acridine orange (AO; Molecular Probes, Eugene, OR, USA) in a phosphate-citrate buffer, pH 6.0. All measurements began 3 min after AO staining. Cells were analyzed by a FACScan (Becton Dickinson, San Jose, CA, USA) equipped with an air-cooled argon ion laser and standard optical filters to collect green and red fluorescence. A total of 10,000 events were accumulated for each measurement. Under these experimental conditions, when excited with a 488 nm light source, AO, when intercalated with double-stranded DNA emits green fluorescence, whereas AO associated with single-stranded DNA emits red fluorescence. Thus, sperm chromatin damage can be quantified by the FCM measurements of the metachromatic shift from green (native, double-stranded DNA) to red (denatured, single-stranded DNA) fluorescence and displayed as red (fragmented DNA) versus green (DNA stainability) fluorescence intensity cytogram patterns. Off-line analysis of the flow cytometric data was carried out by using dedicated software (SCSASoft, SCSA Diagnostics, Brookings, SD, USA). Computer gates are used to determine the proportion of spermatozoa with increased levels of red and green fluorescence, respectively. We have expressed the extent of DNA denaturation in terms of DNA fragmentation index (DFI), which is the ratio of red to total (red plus green) fluorescence intensity (Evenson et al. 2002). The DNA fragmentation index (DFI) value was calculated for each sperm cell in a sample, and the resulting DFI frequency profile for the entire sperm population was obtained (Figure 2A). The normal population of sperm with no detectable DNA damage forms a unimodal distribution. The fraction of sperm with higher red fluorescence intensity represents the population of abnormal sperm with detectable DNA damage. It is expressed as the percentage of sperm showing DNA fragmentation (%DFI). Additionally, we have also considered the fraction of high-DNA-stainable (HDS) cells, which represent immature spermatozoa with incomplete chromatin condensation. The percentage of HDS cells was calculated by setting an appropriate gate on the bivariate cytogram (Figure 2B) and considering those events that exhibit green fluorescence intensity higher than the upper border of the main cluster of the sperm population with a nondetectable %DFI as immature spermatozoa.

For the flow cytometer setup and calibrations, a reference semen sample retrieved from the laboratory repository was used. Samples were measured twice during independent FCM sessions, and the average value was used. Results from the two measurements were highly correlated (DFI, *r* = 0.96; HDS, *r* = 0.96).

### *Determination of CB-153 and* p,p′-*DDE*.

The levels of CB-153 and *p*,*p*′-DDE were determined as previously described (Rignell-Hydbom et al. 2004). Briefly, CB-153 and *p*,*p*′-DDE were extracted from serum by solid-phase extraction (Isolute ENV+; IST, Hengoed, UK) using on-column degradation of the lipids and analysis by gas chromatography mass spectrometry. ^13^C_12_-Labeled CB-153 and ^13^C_12_-labeled *p*,*p*′-DDE were used as internal standards. The selected ion monitoring of *p*,*p*′-DDE was performed at *m*/*z* 318, whereas *m*/*z* 330 was used for the internal standard. The relative standard deviations, calculated from samples analyzed in duplicate at different days, for CB-153 was 7% at 0.6 ng/mL (*n* = 76) and 5% at 1.5 ng/mL (*n* = 37) and for *p*,*p*′-DDE was 12% at 0.6 ng/mL (*n* = 56) and 7% at 2.4 ng/mL (*n* = 50). The detection limits were 0.05 ng/mL for CB-153 and 0.1 ng/mL for *p*,*p*′-DDE. The analyses of CB-153 and *p*,*p*′-DDE were part of the Round Robin inter-comparison program (H. Drexler, Institute and Out-Patient Clinic for Occupational, Social and Environmental Medicine, University of Erlangen-Nuremberg, Erlangen, Germany) with analysis results within the tolerance limits.

### Determination of lipids by enzymatic methods.

Serum concentrations of triglycerides, cholesterol, and phospholipids were determined by enzymatic methods using triglycerides and cholesterol from Boehringer-Mannheim (Mannheim, Germany) and phospholipids from Waco Chemicals (Neuss, Germany). The total lipid concentration in serum was calculated by summation of the amounts of triglycerides, cholesterol, and phospholipids. In these calculations, the average molecular weights of triglycerides and phospholipids were assumed to be 807 and 714. For cholesterol we used an average molecular weight of 571, assuming that the proportion of free and esterified cholesterol in serum was 1:2.

### Hormone analyses.

Serum concentrations of follicle-stimulating hormone (FSH), luteinizing hormone (LH), and estradiol were analyzed with immunofluorometric techniques. The total assay variation coefficients were 2.9, 2.6, and 8.1%, respectively. Serum testosterone and sexual-hormone–binding globulin (SHBG) were measured by commercially available immunoassays. The total assay variation coefficients were 5.5 and 4.6%, respectively. Inhibin B levels were assessed using a specific immunometric method, as previously described, with a detection limit of 15 ng/L and intra-assay and total assay variation coefficients < 7% (Groome et al. 1996).

### Statistical analysis.

In linear regression models, we evaluated the effect of CB-153 and *p*,*p*′-DDE as exposure variables on the outcome variables %DFI and HDS (Table 1). The CB-153 and *p*,*p*′-DDE variables were treated as continuous variables (untransformed and log transformed) as well as categorized variables (into equally sized quintiles). CB-153 and *p*,*p*′-DDE levels correlated strongly (*r* = 0.78), and these variables were not included in the model at the same time. Accordingly, no interaction analyses were performed. Because of the skewed distributions of the %DFI and HDS variables, we also tested whether log transformation of these variables better fulfilled the model assumptions, which was checked by means of residual analysis. As potential confounders, we initially considered age (as a continuous variable), current smoking status (yes/no), abstinence time (as continuous or categorized into 0–2, > 2–4, > 4–6, > 6 days), BMI (as continuous), and levels of testosterone, SHBG, FSH, LH, estradiol, and inhibin B in serum and the testosterone:SHBG ratio. However, there were no associations between the exposure variables and abstinence time (*r*_s_ < 0.03), smoking (mean difference in CB-153 and *p*,*p*′-DDE < 7%), inhibin B (*r*_s_ < 0.06), LH (*r*_s_ < 0.02), testosterone (*r*_s_ < 0.08), or estradiol (*r*_s_ < 0.04). Thus, the above-mentioned variables were excluded from further analyses. The remaining variables were included in the models, one at a time, together with the exposure variable if they showed any association (*p* < 0.20) with %DFI or HDS. If the adjusted effect estimates (i.e., the effect of exposure on %DFI and HDS, respectively) differed < 15% from the crude estimates, we only present the crude estimates.

## Results

There was no significant correlation between the SCSA parameters ln %DFI and ln HDS (*r* = −0.097, *p* = 0.20). Serum levels of CB-153 and *p*,*p*′-DDE ranged from 39 to 1,460 and from 40 to 2,251 ng/g lipid, respectively (medians, 189 and 240 ng/g lipid).

**Figure 3:**
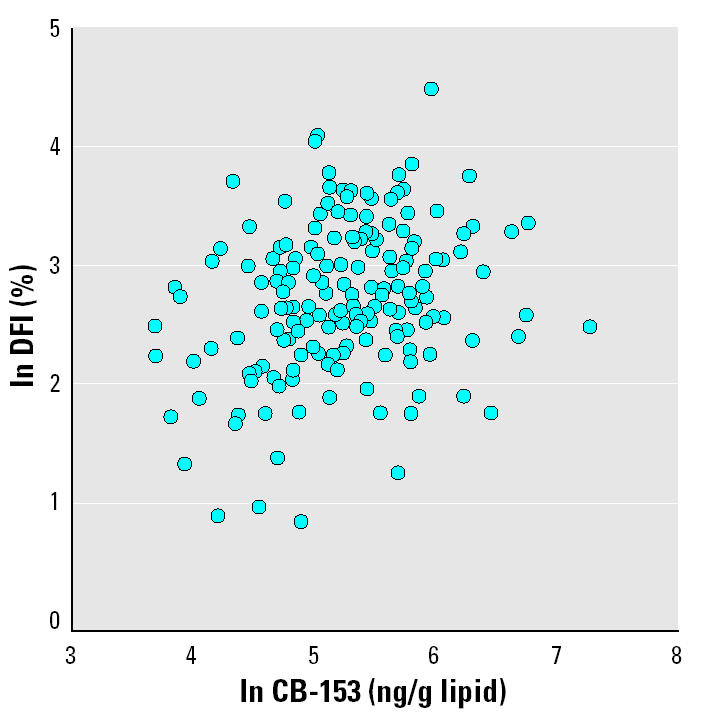
The association between the logarithm of the serum concentration of CB-153 and the logarithm of the DFI (*r* = 0.27, *p* < 0.001).

**Figure 4:**
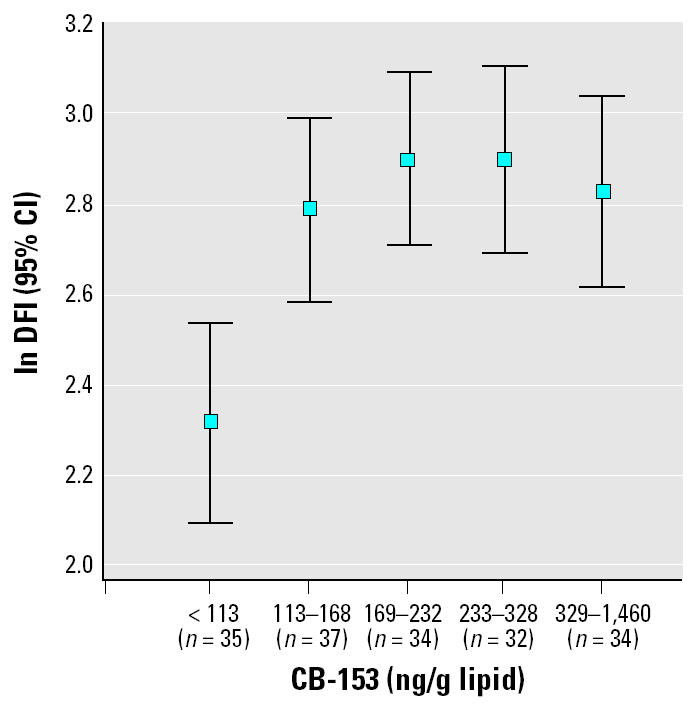
The association between serum concentration of CB-153 (divided into five groups) and the logarithm of DFI (*p* < 0.001).

**Figure 5:**
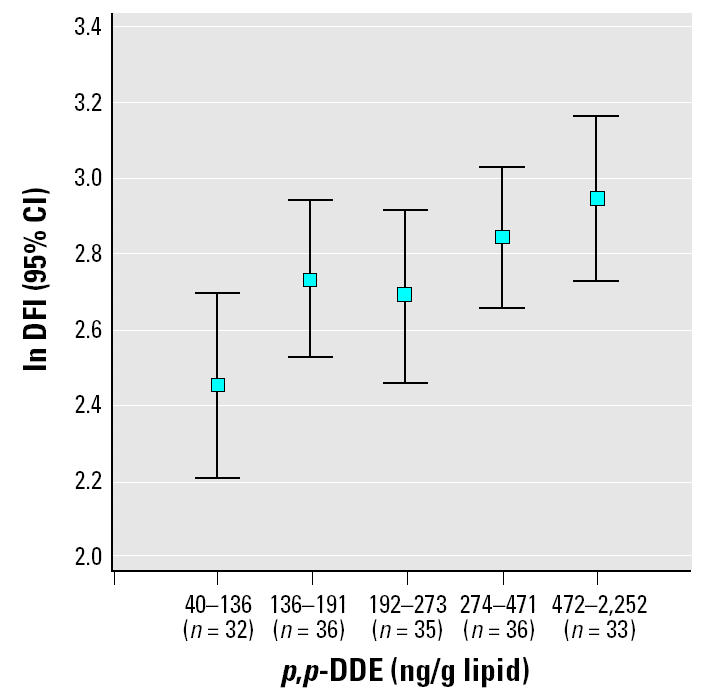
The association between serum concentration of *p*,*p*′-DDE (divided into five groups) and the logarithm of DFI (*p* = 0.10).

In univariate analysis ln CB-153 was associated with ln %DFI (*r* = 0.27, *p* < 0.001; Figure 3). However, when age, which was strongly associated with %DFI, simultaneously was included in the model, this association was no longer significant (*p* = 0.28). On the other hand, when CB-153 was categorized into five equally sized quintiles, there seemed to be an effect (Figure 4). The quintile with the lowest exposure had significantly lower levels of %DFI compared with the other quintiles (*p* < 0.001). This effect remained when age was included in the model (*p* = 0.006). The four highest exposed quintiles (> 113 ng/g lipid) had 41% (95% CI, 11–78) higher %DFI compared with the lowest exposed quintile. None of the other potential confounders changed this estimate more than marginally. In addition, the DFI levels in the four highest exposed quintiles did not differ from each other (all *p*-values > 0.20). Regarding *p*,*p*′-DDE exposure, the pattern was less clear. When age was included in the model (*p* = 0.10), the lowest exposed group (< 136 ng/g lipid) did not significantly differ from the four highest exposed quintiles (22%; 95% CI, −4 to 53; Figure 5). However, the exposure–response pattern for *p*,*p*′-DDE with respect to %DFI was similar as for CB-153 but not statistically significant. Neither CB-153 nor *p*,*p*′-DDE was associated with HDS (all *p*-values > 0.25).

## Discussion

The main result of the present study was a positive association between serum levels of CB-153 and %DFI, indicating that POP exposure might affect sperm DNA integrity. A certain fraction of sperm with DNA breaks is always present in normal ejaculates. Clinical studies using SCSA have, however, demonstrated that the fecundability of a couple is negatively correlated with %DFI when it exceeds 20% (Spano et al. 2000) and becomes negligible for DFI > 30% (Evenson et al. 1999).

Low participation rates and potential selection bias are of great concern in all human semen studies. Both age and demonstrated fertility have an impact on participation rate in semen studies (Larsen et al. 1998a). In the present study, the age distributions as well as the average number of children were very similar among the participants and the nonparticipants. Therefore, we do not suspect that selection bias concerning these factors is of major concern for the interpretation of the results.

A positive correlation (*r* = 0.30, *p* < 0.001) between %DFI and the age of the men enrolled in this study was found. Such an association has been shown previously (Singh et al. 2003; Spano et al. 1998), suggesting less efficient apoptotic mechanisms operating during or after spermatogenesis in aging men. Our study showed, however, an association between serum levels of CB-153 and %DFI, also after adjusting for age. The association was nonlinear, indicating a threshold effect. On the other hand, no associations were found between serum levels of CB-153 and HDS, the SCSA parameter that mirrors the fraction of sperm with defects of the proteic component of the chromatin that characterizes immature spermatozoa (Evenson et al. 2002). The correlation between CB-153 and *p*,*p*′-DDE was high in our study, and the exposure–response pattern for *p*,*p*′-DDE with respect to the outcome variable %DFI showed results pointing into the same direction as for CB-153, but the results were weaker and not statistically significant.

The association between the CB-153 levels and %DFI is in accordance with our recent reports on a correlation between CB-153 and decreased sperm motility (Richthoff et al. 2003; Rignell-Hydbom et al. 2004). A circumstantial evidence supporting the biologic relevance of our observation was that sperm motility was shown to decrease with increasing levels of %DFI (Giwercman et al. 2003). Most of the published SCSA studies, based on infertility patients, report weak, negative significant associations between the %DFI and the parameters from semen quality assessment. In the present study, we found a weak to moderate association between %DFI and sperm motility (*r* = −0.37), which is in close agreement with a study by Giwercman et al. (2003).

There are only two previous studies regarding the association between POP exposure and sperm chromatin damage in humans. In a study carried out in India, 21 infertile men were compared with 32 men with normal semen analyses and evidence of conception (Rozati et al. 2002). Sperm nuclear chromatin integrity was assessed by the chromatin condensation assay using AO staining, and the DNA integrity was monitored under a fluorescent microscope. There was a significant positive correlation between seminal total PCB level and the percentage of single-stranded DNA in sperm. In the other study, carried out in the United States, the neutral single-cell microgel electrophoresis assay (Comet assay) was used to assess DNA integrity in 212 male partners of subfertile couples (Hauser et al. 2003b). The authors did not find any statistically significant associations between sperm DNA damage and serum levels of any individual PCB congeners, sum of PCBs, or *p*,*p*′-DDE, leading to the conclusion that there were no strong relationships. In the American study, CB-153 serum levels ranged from 9 to 421 ng/g lipid (median, 44 ng/g lipid), which is much lower compared with the present study (median, 189 ng/g lipid). The serum levels of *p*,*p*′-DDE were, however, rather similar (median, 254 ng/g lipid) with our present study.

The present study has two advantages compared with the previous ones assessing the association between POP exposure and DNA integrity. First, the study population includes men from the general population and not patients from infertility clinics. Second, SCSA is a computerized technique that ensures a theoretical detection sensitivity advantage, the analysis being based on a large enough number of cells.

A potential mechanism whereby PCBs may produce DNA damage is through hydroxylated PCB metabolites (OH-PCBs), which are found in human serum, in relatively high concentrations (Sjodin et al. 2000). These metabolites can be further oxidized to form semiquinons and quinons (McLean et al. 2000; Schlezinger et al. 1999), which are reactive electrophiles that may induce free-radical–mediated oxidative DNA damage and strand breaks (Li and Trush 1993). PCBs added to human hepatic cell lines increased DNA adduct formation (Borlak et al. 2003). Moreover, PCB quinones inhibited topoisomerase II activity (Srinivasan et al. 2002), which is of key importance for sperm nucleus remodeling.

In addition to more direct mechanisms of toxicity, it has to be considered that experimental data show that several POPs interact with steroid hormone homeostasis and thereby act as “endocrine disruptors” (Bonefeld-Jorgensen et al. 2001; Kester et al. 2002; Portigal et al. 2002; Shevtsov et al. 2003), but the relevance of these effects for sperm chromatin damage is unclear.

In conclusion, we found a statistically significant association between serum levels of CB-153 and %DFI, and a similar tendency although not significant for *p*,*p*′-DDE, in adult men. Further studies are needed to clarify the mechanism responsible for the association between POP exposure and the sperm chromatin integrity.

## Figures and Tables

**Table 1:** Distribution of SCSA results, exposure variables, and potential confounders in 176 Swedish fishermen.

	Mean ± SD	Median	5–95%
SCSA outcome variable			
%DFI	19 ± 12	15	6–39
HDS (%)	10 ± 7	8	4–25
Exposure variables			
CB-153 (ng/g lipid)	233 ± 178	189	63–552
*p,p*′-DDE (ng/g lipid)	334 ± 307	240	80–887
Potential confounder that did not fulfill the inclusion criteria for multivariate models			
Current smoker (%)	23		
Abstinence time (days)	3.8 ± 2.7	3.0	1–9
BMI (kg/m^2^)	27 ± 3.3	27	22–34
Serum LH (IU/L)	2.9 ± 1.2	2.7	1.3–5.2
Serum testosterone (nmol/L)	12.9 ± 5.8	11.7	6.7–22.6
Serum estradiol (pmol/L)	91 ± 43	83	50–158
Serum inhibin (ng/L)	192 ± 69	181	98–308
Serum FSH (IE/L)	4.0 ± 2.2	3.4	1.7–8.5
Serum SHBG (nmol/L)	31.4 ± 12.2	30.9	14.6–54.2
Serum HBG:testosterone	0.44 ± 0.21	0.41	0.23–0.76
Confounder included in the multivariate models			
Age (years)	47 ± 9	48	32–63
